# The Role of PD-L1 Expression in Prediction and Stratification of Recurrent or Refractory Extranodal Natural Killer/T-Cell Lymphoma

**DOI:** 10.3389/fonc.2022.821918

**Published:** 2022-05-10

**Authors:** Li-Min Gao, Yue-Hua Zhang, Xiaoliang Shi, Yang Liu, Junwei Wang, Wen-Yan Zhang, Wei-Ping Liu

**Affiliations:** ^1^ Department of Pathology, West China Hospital of Sichuan University, Chengdu, China; ^2^ Department of Medical Product, OrigiMed, Inc., Shanghai, China

**Keywords:** extranodal natural killer/T-cell lymphoma, relapsed and refractory, PD-L1, risk prognostic model, next-generation sequencing

## Abstract

**Background and Aims:**

The clinical outcome of relapsed and refractory (RR) extranodal natural killer/T-cell lymphoma (ENKTL) is poor. It is necessary to identify RR patients in ENKTL and find novel therapeutic targets to improve the prognosis of patients with RR ENKTL.

**Methods:**

A total of 189 ENKTL patients with effective clinical characteristics were enrolled. Paraffin specimens were collected for PD-L1 expression identification. Kaplan-Meier curve analysis was performed for survival analysis. Whole exome sequencing (WES) was performed for identifying the mutational characterization of RR and effective treatment (ET) patients.

**Results:**

Univariate and multivariate Cox proportional hazards regression analysis showed that negative PD-L1 expression (HR = 1.132, 95% CI = 0.739-1.734, *P* = 0.036) was an independent predictor of poor prognosis in patients with ENKTL. The overall survival (OS) of PD-L1 positive patients was significantly higher than that of PD-L1 negative patients (*P* = 0.009). Then, we added PD-L1 expression as a risk factor to the model of Prognostic Index of Natural Killer Lymphoma (PINK), and named as PINK+PD-L1. The PINK+PD-L1 model can significantly distinguish RR patients, ET patients, and the whole cohort. Moreover, our data showed that PD-L1 expression was lower than 25% in most RR patients, suggesting that RR subtypes may be associated with low expression of PD-L1 (*P* = 0.019). According to the whole exome sequencing (WES), we found that the mutation frequencies of JAK-STAT (*P* = 0.001), PI3K-AKT (*P* = 0.02) and NF-kappa B (*P* < 0.001) pathways in RR patients were significantly higher than those in ET patients.

**Conclusion:**

Patients tend to show RR when PD-L1 expression is lower than 25%. The model of PINK+PD-L1 can stratify the risk of different groups and predict OS in ENKTL patients. The mutational profile of ENKTL patients with RR is different from that of patients with ET.

## Introduction

Extranodal natural killer/T-cell lymphoma (ENKTL) is an Epstein–Barr virus (EBV)-associated aggressive lymphoma (1). It is not only endemic in East Asia, Central America, and South America, but also sporadic in Western countries (2). Although L-asparaginse regimens have significantly improved the prognosis and are most commonly used initial therapy, L-asparaginse regimens still fail in 20%–40% of cases (3–6). To date, there is still a lack of effective indicators for predicting relapsed and refractory (RR) ENKTL patients during early diagnosis and further risk stratification of RR patients has not been reported yet.

Immune checkpoint inhibitors (ICIs) of the programmed death 1 (PD-1)/programmed death ligand 1 (PD-L1) pathway is an extremely active area of laboratory and clinical investigation and evidence demonstrates that ICIs can benefit patients with advanced cancer from both overall survival (OS) benefit and durable response (7, 8). Many clinical trials of PD-1/PD-L1 blockade, in the treatment of solid tumors and lymphomas, have been conducted and some surprising results have been achieved (9–12). However, the relationship of PD-L1 expression status and prognosis in ENKTL remains controversial (13–25), There are few studies on the regulation of PD-L1 expression (8, 17, 26, 27), as well as the reports on the relationship of PD-L1 expression status and prognosis in RR ENKTL (17, 26). In this study, we investigated the PD-L1 expression in 189 cases, stratified ENKTL according to the expression of PD-L1 expression, analyzed the relationship between PD-L1 expression and RR and ET, and preliminarily explored gene mutations in the pathways related with *CD274*, which encodes PD-L1 protein.

## Materials and Methods

### Patients

A total of 189 ENKTL patients were collected from Department of Pathology, West China Hospital of Sichuan University from 2009 to 2019. All cases were reviewed by experienced pathologists according to the World Health Organization (WHO) classification of hematopoietic and lymphoid tissues (28). All cases were divided into RR group and effective treatment (ET) group according to the NCCN Guidelines Insights: T-Cell Lymphomas, Version 1.2021 (29). Being relapsed was defined as the emergence of new lesions after achieving complete response (CR) within six months and refractory was defined as partial response (PR) not achieved after chemotherapy. Being ET was defined as CR or PR was achieved after 4 cycles of chemotherapy, with no disease recurrence or progression within 6 months. The response was evaluated according to the Lugano response criteria for non-Hodgkin lymphoma (30). OS was defined as the number of days from the date of diagnosis to the date of death or final follow-up.

Anonymous data regarding age, gender, Eastern Cooperative Oncology Group performance status (ECOG PS), Prognostic Index of Natural Killer Lymphoma (PINK), lesion site, Ann Arbor stage, lactate dehydrogenase (LDH) level, B symptoms, asparaginase usage, and survival time were retrospectively obtained from the patients’ medical records and telephone follow-ups. All patients were followed up from the date of diagnosis to December 25, 2020. This study was approved by the Medical Ethics Committee of West China Hospital of Sichuan University (number: WH-20201220). All recruited patients gave written informed consent in accordance with the Declaration of Helsinki.

### PD-L1 Expression Analysis

**Figure 1:**
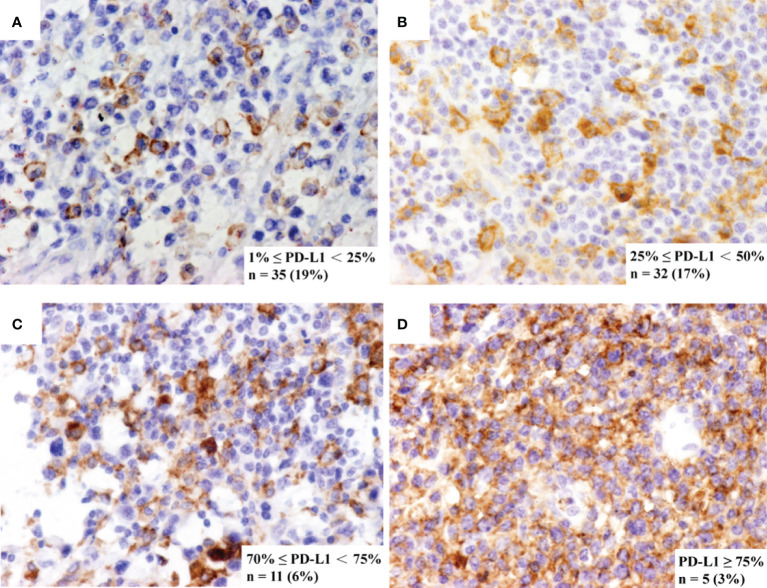
Representative immunohistochemistry staining of PD-L1. The immunohistochemistry panel shows representative figures of **(A)** 1% ≤ PD-L1 < 25%, detected in 19% of cases, **(B)** 25% ≤ PD-L1 < 50%, detected in 17% of cases, **(C)** 50% ≤ PD-L1 < 75%, detected in 6% of cases and **(D)** PD-L1 ≥ 75%, detected in 3% of cases.

Immunohistochemistry (IHC) staining of PD-L1 (SP142, ZHONGSHAN, Beijing, CHINA) was performed by using EliVision method (31). PD-L1 expression was evaluated on tumor cells by certified pathologists. PD-L1 positive status was defined as the presence of membrane staining of any intensity in 1% or more of tumor cells. Representative staining, case frequency, and number information for each marker are shown in **Figure 1**.

### DNA Isolation

Considering DNA degradation of the formalin-fixed paraffin-embedded (FFPE) tissue specimens, 21 RR and 10 ET FFPE tissue specimens from the past 3 years were selected for further whole exome sequencing (WES). Genomic DNA of these specimens was extracted by using QIAamp DNA FFPE Tissue Kit (Qiagen Inc., Valencia, CA, USA).

### Next-Generation Sequencing and Bioinformatics Analysis

According to the manufacturer’s instructions, a minimum of 50ng DNA was used for library preparation and sequencing on Nextseq500 (Illumina, Inc., CA, USA). The quality and size of the fragments were assessed using a Qubit 2.0 Fluorimeter with the dsDNA high sensitivity assay kit (Life Technologies; Thermo Fisher Scientific, CA, USA). WES was performed with a mean depth of 500× by using the Sure-Select Human All Exon V6 kit (Agilent, Santa Clara, CA, USA) on tumor biopsies and matched peripheral-blood mononuclear cell samples. Genomic alterations (GAs) including Single nucleotide variants (SNVs), insertion-deletion polymorphisms (Indels), copy number variation (CNV), were identified by using MuTect (v1.17), PINDEL (v2.04), and Control-FREEC (v9.4), respectively.

### Statistical Analysis

Continuous biologic variables were dichotomized and frequency tables were analyzed using the chi-squared (χ^2^) test. Estimates of hazard ratios (HRs) and 95% confidence intervals (CIs) were calculated using a Cox proportional hazards regression model. Kaplan-Meier survival analysis and the log-rank test was used to examine differences in OS. All statistical analyses were performed using SPSS software version 26 (SPSS Inc., Chicago, IL, USA). *P* < 0.05 was considered statistically significant.

## Results

### Patient Characteristics

The cohort included 189 ENKTL patients, of whom 136 had complete treatment information. There were 130 nasal cases and 59 extra-nasal cases, with male to female ratio of 2.26:1. 64 (33.86%) patients present with B symptoms; 131 (69.32%) patients were at stage I-II and 58 (30.68%) were at stage III-IV. According to the NCCN guideline, 88 ENKTL patients were classified as ET group and 48 ENKTL patients were classified as RR group. The remaining 53 patients were excluded from the classification due to their incomplete treatment information. In the ET group, there were 56 (63.64%) men and 32 (36.36%) women, of which 66 (75.00%) were stage I-II and 22 (25.00%) were stage III-IV. While in RR group, there were 38 (79.17%) patients were men and 10 (20.83%) women, of which 24 (50.00%) were stage I-II and 24 (50.00%) were stage III-IV. The clinical characteristics of this cohort are shown in **Table 1**

**Table 1:** Clinical and pathological features of 189 ENKTL cases.

Clinical and pathological features	Total	ET (n = 88)	RR (n = 48)
Age			
≤ 60	163 (86.24%)	74 (84.09%)	41 (85.42%)
> 60	26 (13.76%)	14 (15.91%)	7 (14.58%)
Gender			
Male	131 (69.31%)	56 (63.64%)	38 (79.17%)
Female	58 (30.69%)	32 (36.36%)	10 (20.83%)
ECOG PS (n = 153)			
≤ 2	122 (79.74%)	76 (86.36%)	31 (64.58%)
> 2	31 (20.26%)	12 (13.64%)	17 (35.42%)
PINK (n = 153)			
Low risk	71 (46.41%)	50 (56.82%)	15 (31.25%)
Intermediate risk	32 (20.91%)	21 (23.86%)	6 (12.50%)
High risk	50 (32.68%)	17 (19.32%)	27 (56.25%)
Lesion site			
Nasal	130 (68.78%)	68 (77.27%)	24 (50.00%)
Extra-nasal	59 (31.22%)	20 (22.73%)	24 (50.00%)
Ann Arbor stage			
I	70 (37.04%)	37 (42.05%)	10 (20.83%)
II	61 (32.28%)	29 (32.95%)	14 (29.17%)
III	6 (3.17%)	3 (3.41%)	1 (2.08%)
IV	52 (27.51%)	19 (21.59%)	23 (47.92%)
LDH level (IU) (n = 161)			
≤ 220	99 (61.49%)	61 (69.32%)	23 (37.92%)
> 220	62 (38.51%)	27 (30.68%)	25 (52.08%)
B symptoms			
No	125 (66.14%)	62 (70.45%)	28 (58.33%)
Yes	64 (33.86%)	26 (29.55%)	20 (41.67%)
Chemotherapy (n = 163)			
With asparaginase	103 (63.19%)	66 (75.00%)	31 (64.58%)
Without asparaginase	60 (36.81%)	22 (25.00%)	17 (35.42%)
Radiotherapy			
Yes	119 (62.96%)	71 (80.68%)	31 (64.58%)
No	70 (37.04%)	17 (19.32%)	17 (35.42%)
PD-L1			
Negative	106 (56.08%)	34 (38.64%)	25 (52.09%)
1% ≤ PD-1 < 25%	32 (16.93%)	16 (18.18%)	12 (25.00%)
25% ≤ PD-1 < 50%	35 (18.52%)	26 (29.55%)	7 (14.58%)
50% ≤ PD-1 < 75%	11 (5.82%)	10 (11.36%)	1 (2.08%)
75% ≤ PD-1 ≤ 100%	5 (2.65%)	2 (2.27%)	3 (6.25%)
Survival state			
Survive	50 (26.46%)	36 (40.91%)	7 (14.58%)
Dead	139 (73.54%)	52 (59.09%)	41 (85.42%)

ENKTL, extranodal NK/T-cell lymphoma, nasal type; ECOG PS, Eastern Cooperative Oncology Group performance status; PINK, Prognostic Index of Natural Killer Lymphoma; ET, effective treatment; RR, relapsed or refractory; LDH, lactate dehydrogenase.

### Association Analysis Between Clinicopathological Characteristics and PD-L1 Expression and Prognosis in 189 ENKTL Patients

Univariate Cox proportional hazards regression analysis demonstrated that ECOG PS > 2 (HR = 10.878, 95% CI = 6.522-18.143, *P* < 0.001), PINK high-risk (HR = 4.125, 95% CI = 2.603-6.537, *P* < 0.001), extra-nasal (HR = 2.268, 95% CI = 1.595-3.225, *P* < 0.001), Ann Arbor stage III-IV (HR = 1.976, 95% CI = 1.380-2.830, *P* < 0.001), LDH > 220 IU (HR = 3.138, 95% CI = 2.148-4.584, *P* < 0.001), no asparaginase usage (HR = 3.356, 95% CI = 2.293-4.911, *P* < 0.001), and PD-L1 negative expression (HR = 1.572, 95% CI = 1.116-2.214, *P* = 0.009) was associated with poor OS (**Table 2**). Multivariate Cox proportional hazards regression analysis demonstrated that PD-L1 negative expression was independently associated with an increased case fatality rate (HR = 1.132, 95% CI = 0.739-1.734, *P* = 0.036). In addition, ECOG PS > 2 (HR = 4.086, 95% CI = 1.883-8.866, *P* < 0.001), PINK high-risk (HR = 1.932, 95% CI = 0.993-3.759, *P* = 0.049), LDH > 220 IU (HR = 2.428, 95% CI = 1.589-3.710, *P* < 0.001) and no asparaginase usage (HR = 1.883, 95% CI = 1.158-3.061, *P* = 0.011) were also independently associated with an unfavorable prognosis (**Table 3**).

**Table 2:** Univariate analysis of prognostic factors in patients with 189 ENKTL.

	Univariate analysis		
Factor	HR	95% CI	*P*
Age			
≤ 60	1.000		
> 60	1.158	0.693 - 1.934	0.576
Gender			
Male	1.000		
Female	0.919	0.635 - 1.331	0.656
ECOG PS			
≤ 2	1.000		
> 2	10.878	6.522 - 18.143	< 0.001
PINK			
Low	1.000		
Intermediate	1.282	0.742 - 2.215	0.374
High	4.125	2.603 - 6.537	< 0.001
Lesion site			
Nasal	1.000		
Extra-nasal	2.268	1.595-3.225	< 0.001
Ann Arbor stage			
I-II	1.000		
III-IV	1.976	1.380 - 2.830	< 0.001
LDH level (IU)			
≤ 220	1.000		
> 220	3.138	2.148 - 4.584	< 0.001
B symptoms			
No	1.000		
Yes	1.267	0.897 - 1.790	0.179
Asparaginase usage			
Yes	1.000		
No	3.356	2.293 - 4.911	< 0.001
PD-L1			
Positive	1.000		
Negative	1.572	1.116 - 2.214	0.009

P were determined by univariate Cox proportional hazards regression analysis. OS, overall survival; ENKTL, extranodal NK/T-cell lymphoma, nasal type; HR, hazard ratio; CI, confidence interval; ECOG PS, Eastern Cooperative Oncology Group performance status; PINK, Prognostic Index of Natural Killer Lymphoma; LDH, lactate dehydrogenase.

**Table 3:** Multivariate analysis of prognostic factors in patients with 189 ENKTL.

		Multivariate analysis	
Factor	HR	95% CI	*P*
ECOG PS			
≤ 2	1.000		
> 2	4.086	1.883 - 8.866	< 0.001
PINK			
Low	1.000		
Intermediate	1.351	0.766 - 2.382	0.298
High	1.932	0.993 - 3.759	0.049
LDH level (IU)			
≤ 220	1.000		
> 220	2.428	1.589 - 3.710	< 0.001
Asparaginase usage			
Yes	1.000		
No	1.883	1.158 - 3.061	0.011
PD-L1			
Positive	1.000		
Negative	1.132	0.739 - 1.734	0.036

P were determined by multivariate Cox proportional hazards regression analysis. OS, overall survival; ENKTL, extranodal NK/T-cell lymphoma, nasal type; HR, hazard ratio; CI, confidence interval; ECOG PS, Eastern Cooperative Oncology Group performance status; PINK, Prognostic Index of Natural Killer Lymphoma; LDH, lactate dehydrogenase.

**Figure 2:**
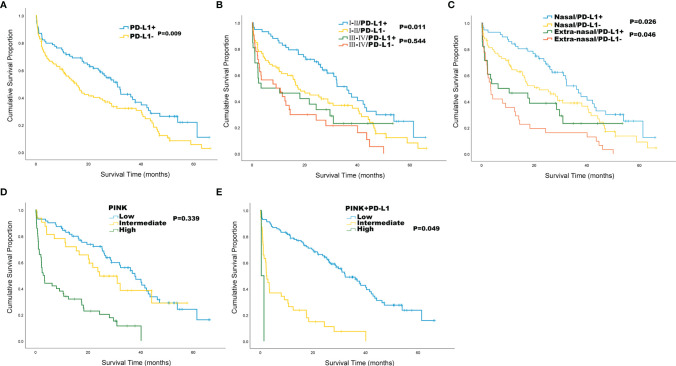
Kaplan-Meier survival analysis for the whole cohort of 189 patients with ENKTL. **(A)** Overall survival (OS) by PD-L1 status; **(B)** OS by Ann Arbor stage and PD-L1 status; **(C)** OS by lesion site and PD-L1 status; **(D)** OS by PINK; **(E)** OS by PINK+PD-L1.

Compared with PD-L1 negative patients, PD-L1 positive patients had better ECOG PS (*P* = 0.004) and better survival status (*P* = 0.010) (**Table 4**). According to the survival curve analysis, the OS in PD-L1 positive patients was significantly longer than that in PD-L1 negative patients (30.9 vs 15.5 months, *P* = 0.009; **Figure 2A**). When stratified by Ann Arbor stage, we found that the median OS of PD-L1 positive patients was significantly longer than that of PD-L1 negative patients in Ann Arbor stage I-II group (36.1 vs 17.5 months, *P* = 0.011, **Figure 2B**) in PD-L1 negative patients; while in Ann Arbor stage III-IV group, there is no significant difference in median OS between PD-L1 positive and PD-L1 negative patients (*P* = 0.544; **Figure 2B**). When stratified by lesion site, our results showed that the median OS in nasal group was 36.1 versus 22.0 months (*P* = 0.026; **Figure 2C**), and the median OS in extra-nasal group was 6.3 versus 3.5 months (*P* = 0.046; **Figure 2C**) in PD-L1 positive and PD-L1 negative patients, respectively.

**Table 4:** Clinical and pathological features of the PD-L1 positive compared to PD-L1 negative in 189 ENKTL cases.

Clinical and pathological features	PD-L1 positive	PD-L1 negative	*P*
Age			0.850
≤ 60	72	91	
> 60	12	14	
Gender			0.573
Male	60	71	
Female	24	34	
ECOG PS (n = 153)			0.004
≤ 2	71	51	
> 2	9	22	
PINK (n = 153)			0.176
Low risk	42	29	
Intermediate risk	17	15	
High risk	21	29	
Lesion site			0.574
Nasal	56	74	
Extra-nasal	28	31	
Ann Arbor stage			0.763
I	34	36	
II	24	37	
III	3	3	
IV	23	29	
LDH level (IU) (n = 161)			0.174
≤ 220	54	45	
> 220	27	35	
B symptoms			0.287
No	59	66	
Yes	25	39	
Treatment response (n = 136)			0.100
ET	55	33	
RR	23	25	
Survival state			0.010
Survive	30	20	
Dead	54	85	

P were determined by Chi-squared (χ^2^) test. ENKTL, extranodal NK/T-cell lymphoma, nasal type; ECOG PS, Eastern Cooperative Oncology Group performance status; PINK, Prognostic Index of Natural Killer Lymphoma; ET, effective treatment; RR, relapsed or refractory; LDH, lactate dehydrogenase.

The PINK score included 4 risk factors of age > 60, Ann Arbor stage III-IV, distant lymph-node involvement, and non-nasal type disease. Since PINK can identify high-risk patients, we tried to stratify patients by using PINK to separate the risk of ENKTL patients and the results showed that patients with low and intermediate PINK cannot be separated by OS (P = 0.339, **Figure 2D**). Then, we designed a novel PINK system named PINK+PD-L1 by adding PD-L1 as a risk factor. The PINK+PD-L1 shows a score standard of low risk (0-2 risk factors), intermediate risk (3-4 risk factors), and high-risk (5 risk factors), and can well distinguish ENKTL with median OS of 32.3 months, 2.3 months, and 0.4 months in low, intermediate, and high-risk stratification, respectively (**Figure 2E**).

### Analysis of Relapsed or Refractory and Effective Treatment ENKTL Patients

**Figure 3:**
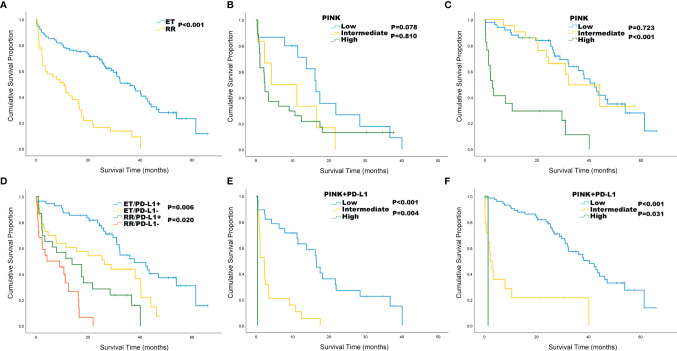
Kaplan-Meier survival analysis for ENKTL patients in relapsed or refractory (RR) and effective treatment (ET) group. **(A)** Overall survival (OS) by treatment response; **(B)** OS of RR group by PINK; **(C)** OS of ET group by PINK; **(D)** OS by treatment response and PD-L1 status. **(E)** OS of RR group by PINK+ PD-L1; **(F)** OS of ET group by PINK+ PD-L1.

We performed Kaplan-Meier survival analysis to compare the clinical indicators between RR and ET in ENKTL patients. Our results showed that the OS of RR group was significantly shorter than that of ET group (10.7 vs 36.1 months, *P* < 0.001; **Figure 3A**). We tried to stratify the risk of ENKTL patients by using PINK standard, but failed to stratify the risk (*P* = 0.078, *P* = 0.810, respectively; **Figure 3B**) in RR group. While in ET group, patients with low and intermediate PINK are poorly separated by OS (*P* = 0.723, **Figure 3C**). The patients were further divided into PD-L1 positive group and PD-L1 negative group and results showed that the OS of PD-L1 negative patients were worse than that of PD-L1 positive patients in both RR (4.2 vs 13.9 months, *P* = 0.020) and ET (26.3 vs 37.8 months, *P* = 0.006) groups (**Figure 3D**). However, it is difficult to distinguish RR group with other PD-L1 thresholds (**Supplementary Figure 1**). When the risk of RR and ET groups was stratified with PINK+PD-L1, it was well separated as low, intermediate, and high-risk in RR group, with the median OS of 16.5, 2.2, and 0.4 months, respectively (**Figure 3E**). Kaplan-Meier survival analysis also showed the statistically significantly improved separation of low and intermediate risk by OS in ET group (**Figure 3F**).

In addition, we also analyzed the correlation between PD-L1 expression and ENKTL subtypes (RR and ET). Interestingly, we found that patients tend to show RR when PD-L1 expression lower than 25% (*P* = 0.019; **Table 5**).

**Table 5:** Different cut-off values of PD-L1 in relapsed or refractory (RR) and effective treatment (ET) patients with ENKTL.

	Negative	Positive	*P*	< 25%	≥ 25%	*P*	< 50%	≥ 50%	*P*	< 75%	≥ 75%	*P*
RR	25	23	0.100	37	11	0.019	44	4	0.418	45	3	0.345
ET	33	55		50	38		76	12		86	2	

P were determined by Chi-squared (χ^2^) test. RR, relapsed or refractory; ET, effective treatment.

### Mutational Characterization of PD-L1 Related Pathways in Relapsed or Refractory and Effective Treatment ENKTL Patients

**Figure 4:**
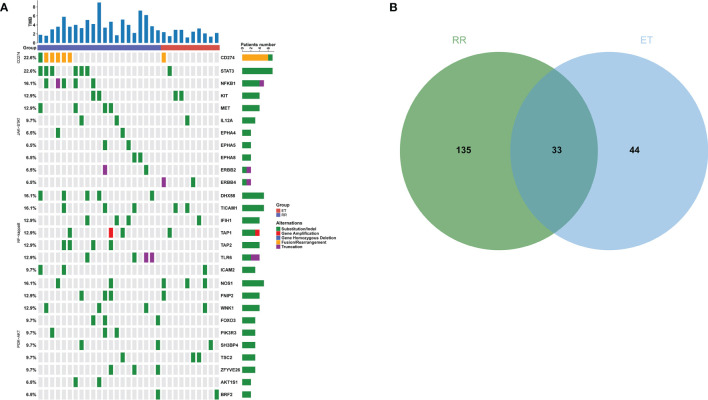
Mutational Profile of Relapsed or Refractory (RR) and Effective Treatment (ET) ENKTL Patients. **(A)** The distribution and frequency of genetic alterations in RR and ET ENKTL patients. The types of mutation are labeled in different colors. **(B)** Venn diagram depicting the number of genes exclusive or shared between RR and ET ENKTL patients.

Based on the WES detection, we analyzed PD-L1 related mutations, including the *CD274* encoding PD-L1, and 607 genes in three pathways related to *CD274* previously reported in literature (8, 16, 32). The mutation frequency of *CD274* in RR group was 28.57% (6/21, 5 rearrangement mutations and 1 deletion mutation) and 10.00% (1/10, rearrangement mutation) in RR group and ET group, respectively (*P* = 0.379). In the RR group, a total of 253 mutations in 165 genes were identified. The most common mutated genes were *CD274*, *STAT3*, *DHX58* and *NFKB1* (**Figure 4A**). While in the ET group, 87 mutations from 77 genes were identified and *NOS1* was the most frequently mutated gene (**Figure 4A**). Mutations in 131 genes such as *AKT1*, *MET*, and *TP53* were exclusively detected in RR group and 33 mutated genes were shared in both RR and ET group (**Figure 4B**). Compared with ET group, the mutational frequency of JAK-STAT (*P* = 0.001), PI3K-AKT (*P* = 0.02), and NF-kappa B (*P* < 0.001) pathways were significantly more frequent in RR group (**Table 6**).

**Table 6:** Analysis of three pathways related genes in relapsed or refractory (RR) and effective treatment (ET) patients.

Pathway	JAK-STAT			PI3K-AKT			NF-kappa B		
	Mutation	No mutation	*P*	Mutation	No mutation	*P*	Mutation	No mutation	*P*
RR	45	143	0.001	39	53	0.002	96	231	< 0.001
ET	20	168		19	73		46	281	

P were determined by Chi-squared (χ^2^) test. RR, relapsed or refractory; ET, effective treatment.

## Discussion

This is the largest study on PD-L1 expression status and corresponding prognostic features in ENKTL to date. PD-L1 expression is a new therapeutic target in cancer immunotherapy. Recent studies have suggested that PD-L1 expression could be a main factor in cancer progression through inhibiting anti-cancer immune response (33, 34). Indeed, many solid tumor cells can escape the host immune system by expressing PD-L1, and then activating immunosuppressive signals (35, 36). Lymphoma is a malignancy of immune system cells and the role of the PD-L1 in lymphoma is more complicated. In ENKTL, there have been many studies on the expression of PD-L1 protein (**Table 7**) (13, 15, 17–25). The maximum sample size of these studies is only 81 cases and the cut off values and the prognostic results are varied between studies. Our data suggest that PD-L1 expression is an independent predictor of the better prognosis in ENKTL patients. The PD-1/PD-L1 pathway can inhibit interleukin-2 (IL-2) and interferon-γ, and IL-2 is important for the proliferation and maintenance of ENKTL cells. Therefore, the biological process of tumor cells mediated by PD-L1 in ENKTL may be associated with the decrease of cytokine release (13, 37, 38). PD-L1 may lead to the depletion of cytokines involved in the survival and growth of tumor cells, resulting in antitumor effects in ENKTL (37). In addition, it has been reported that PD-L1 expressed on ENKTL tumor cells may transmit inhibitory signals in tumor cells, which may be associated with good prognosis (37). However, more experience is required to confirm this conclusion and explore its potential mechanism.

**Table 7:** Summary of the PD-L1 cut off values and prognostic significance of ENKTL cases from literatures and our study.

Author	Cases	PD-L1		
		Cut off value	Positive rate	Prognostic significance
Our study	189	Positive	83/189	Better
Zhang et al.(18)	51	10%	39/51	Better
Muhamad et al.(19)	49	Positive	30/49	Worse
Lam et al.(20)	81	10%	44/81	Worse
Kim et al.(17)	21	10%	11/21	Better
Cai et al.(22)	7	5%	6/7	–
Zeng et al.(21)	42	5%	25/42	Worse
Kataoka et al.(23)	19	5%	6/19	–
Li et al.(24)	12	5%	7/12	–
Kim et al.(25)	41	–	–	–
Jo et al.(15)	79	5%	63/79	No difference
Kim et al.(13)	73	10%	41/73	Better

ENKTL, extranodal natural killer/T-cell lymphoma; -, not mention.

In this study, correlation analysis showed that ENKTL patients with PD-L1 < 25% were more likely to develop into RR. To date, there are few studies on PD-L1 expression in RR ENKTL patients. Contrary to our study, Kim et al. and Lim et al. show that PD-L1 expression is high in RR ENKTL patients (17, 26).The expression status of PD-L1 may depend on the corresponding genomic mutations (39). For example, EBV-driven LMP1 can induce the expression of PD-L1 (16, 40, 41). In the studies of Kim et al. and Lim et al., PD-L1 expression was identified after chemotherapy, which may be more related to EBV-DNA level. In addition, our results supported that ENKTL patients with PD-L1 ≥ 25% are more likely to be ET. These patients may be relatively sensitive to chemotherapy, which is consistent with the results of Feng et al. (42). Feng et al. show that BRAF V600E mutation may induce the expression of PD-L1 and then increase chemotherapy-induced apoptosis by inducing BIM and BIK proteins in colon cancer 40.

**Figure 5:**
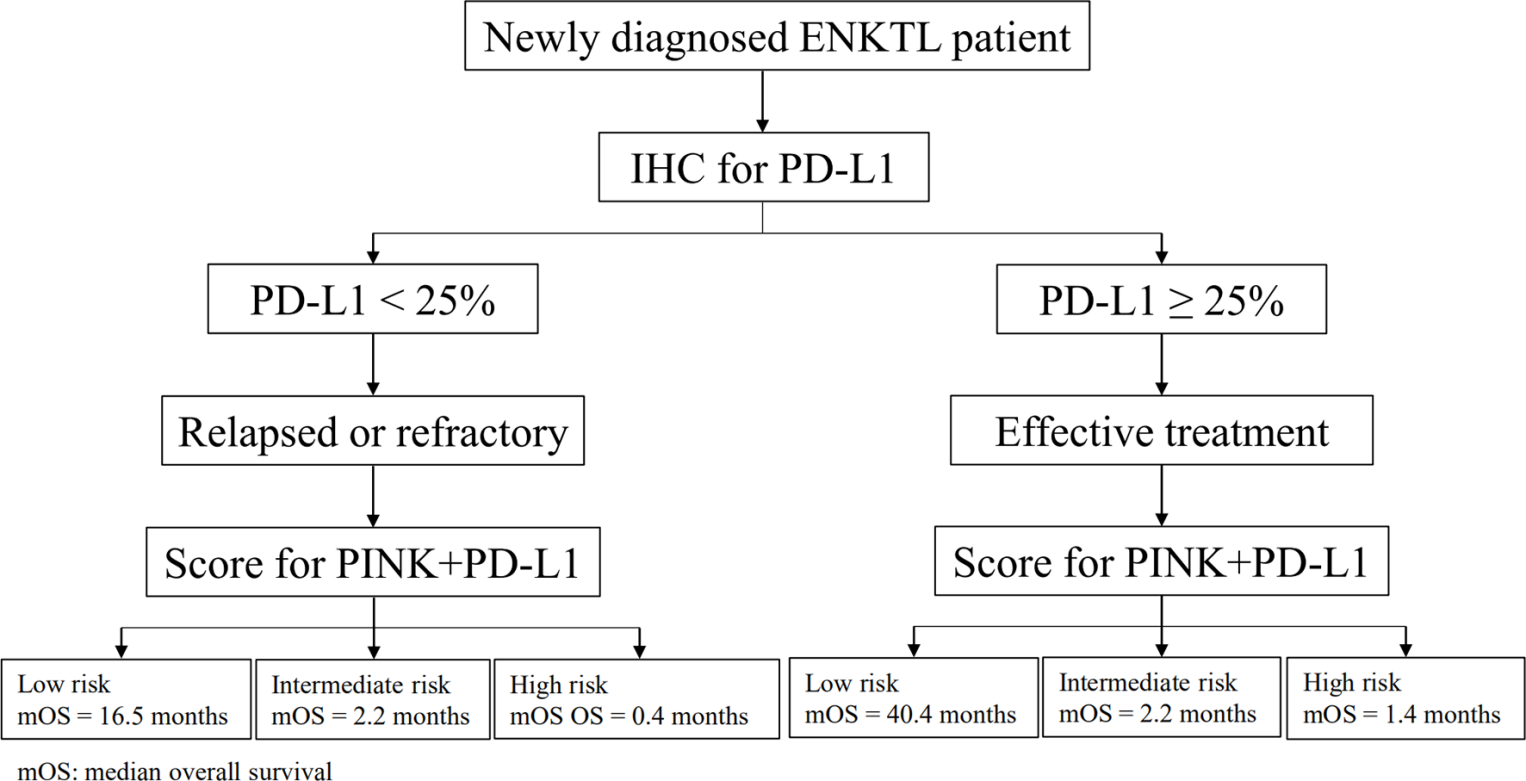
The flow chart for establishment the median OS of different patients.

PINK is a prognostic model based on non-anthracycline-based regimens to predict outcomes in ENKTL (43). However, there is still no relevant report to clarify whether the PINK model can carry out risk assessment in RR and ET patients. In our study, the PINK model failed to stratify the risk of RR patients and could not distinguish low-risk and intermediate-risk of ET patients, as well as the whole cohort. The reason may be that there are too many high-risk factors in RR patients, which is not conducive PINK to clearly stratify. Many other ENKTL prognostic models had been explored. For example, the International Prognostic Index (IPI) from the International Peripheral T-Cell Lymphoma Project (44) and the Korean Prognostic Index (KPI) from a Korean multicenter study (45) were explored for patients who were primarily treated with anthracycline-based regimens; and the nomogram-revised risk index (NRI) from a Chinese multicenter study (46) was designed for predicting survival of early-stage patients who require radiotherapy. However, none of them are suitable for ENKTL RR patients. Considering that PD-L1 can further stratify ET, RR patients, and the whole cohort, we combined PD-L1 with PINK to develop a new prognostic model PINK+PD-L1. This model can stratify the risk of RR patients and can also be used in ET patients and the whole cohort. We summarized the flow chart of estimating the median OS of different patients based on PINK+PD-L1 (**Figure 5**), which can assist clinicians in judging the prognosis and risk stratification of initially diagnosed ENKTL patients and then assist to select the appropriate treatment strategy.

A previous study showed that 21.1% (4/19) RR ENKTL patients had PD-L1 structural rearrangement (26). Similar features were found in our study, showing that the coding gene *CD274* of PD-L1 was rearranged in 23.81% (5/21) RR ENKTL.*CD274* rearrangement has also been reported in adult T-cell leukemia/lymphoma and primary mediastinal large B-cell lymphoma (47, 48). These results support that PD-L1 rearrangement may be an important mutation type of RR ENKTL and play an important role in the occurrence and development of RR ENKTL.

In addition, we also found that the mutation frequency of JAK-STAT, PI3K-AKT, and NF-kappa B signal pathway genes in the RR group was significantly higher than that in the ET group, suggesting the important role of these three signal pathway genes in RR ENKTL. The activation of JAK-STAT, PI3K-AKT, and NF kappa B signaling pathways can regulate the proliferation of tumor cells in ENKTL (49–52), and the crosstalk of these pathways in lymphoma has also been reported (53). Together, these may explain the poor prognosis of RR ENKTL patients.

## Conclusions

In conclusion, PD-L1 positive is a better prognostic factor in ENKTL. Patients tend to show RR when PD-L1 expression lower than 25%. We explored a new prognostic model of PINK+PD-L1, which can stratify the risk of different groups and predict OS in ENKTL patients. Meanwhile, our results suggested that the rearrangement of *CD274* (PD-L1) and the frequent mutation of JAK-STAT, PI3K-AKT, and NF kappa B signal pathway genes play an important role in the occurrence and development of RR ENKTL.

## Data Availability Statement

The datasets presented in this study can be found in online repositories. The names of the repository/repositories and accession number(s) can be found below: China National Genebank, http://db.cngb.org/cnsa/variant/CNP0002517_8292c901/reviewlink/.

## Ethics Statement

The studies involving human participants were reviewed and approved by the Medical Ethics Committee of West China Hospital of Sichuan University (number: WH-20201220). The patients/participants provided their written informed consent to participate in this study.

## Author Contributions

L-MG and Y-HZ made substantial contributions to data collection and was a major contributor in writing the manuscript. Y-HZ analyzed and interpreted the data contributed to manuscript preparation. XS, YL, and JW interpreted the data, and revised the manuscript. W-YZ was responsible for the acquisition of data and institutional review board application, and gave final approval for the version to be published. W-PL agreed to be accountable for all aspects of the work in ensuring that questions related to the accuracy or integrity of any part of the work are appropriately investigated and resolved. All authors contributed to the article and approved the submitted version.

## Funding

This study was supported by the national natural science foundation of China (81900197) and Science and Technology Program of Sichuan Province (2020YJ0104).

## Conflict of Interest

Author XS, JW, and YL were employed by OrigiMed Inc.

The remaining authors declare that the research was conducted in the absence of any commercial or financial relationships that could be construed as a potential conflict of interest.

## Publisher’s Note

All claims expressed in this article are solely those of the authors and do not necessarily represent those of their affiliated organizations, or those of the publisher, the editors and the reviewers. Any product that may be evaluated in this article, or claim that may be made by its manufacturer, is not guaranteed or endorsed by the publisher.

## Supplementary Material

Supplementary Table 1 Three pathways related genes used for next-generation sequencing.

Supplementary Figure 1 Kaplan-Meier survival analysis for relapsed or refractory (RR) ENKTL patients with different cut-off values of PD-L1. **(A)** Overall survival (OS) by PD-L1 cut-off value of 25%; **(B)** OS by PD-L1 cut-off value of 50%; **(C)** OS by PD-L1 cut-off value of 75%.
