# Supplementary material for: The Role of PD-L1 Expression in Prediction and Stratification of Recurrent or Refractory Extranodal Natural Killer/T-Cell Lymphoma
Source: Front Oncol. 2022 May 10;12:821918. doi: 10.3389/fonc.2022.821918 (PMC9128790; doi:10.3389/fonc.2022.821918)
Supplement: Supplementary Table 1 — Three pathways related genes used for next-generation sequencing. [file Table_1.pdf]

Supplementary Table 1. Three pathways related genes used for next-generation sequencing.

Pathway	Genes
JAK-STAT<br>n = 188	*AKT1, ALK, BCL2, BCL2L1, BIRC5, CCL11, CCL13, CCL15, CCL17, CCL18, CCL19, CCL2, CCL20, CCL21, CCL25, CCL3, CCL4, CCL5, CCL7, CCL8, CCND1, CD2, CD4, CD79A, CD79B, CD80, CDK5, CSF1R, CXCL12, CXCL9, CXCR4, EGF, EGFR, EPHA1, EPHA2, EPHA3, EPHA4, EPHA5, EPHA7, EPHA8, EPO, EPOR, ERBB2, ERBB4, EZH2, F2R, FGF1, FGF10, FGF16, FGF17, FGF19, FGF2, FGF3, FGF4, FGF5, FGF6, FGF7, FGF8, FGF9, FGFR1, FGFR2, FGFR3, FGFR4, FLT1, FLT3, FLT4, GH1, GHR, GRB2, HGF, IFNA1, IFNA2, IFNAR1, IFNAR2, IFNB1, IFNG, IFNGR1, IGF1R, IL10, IL10RA, IL10RB, IL11, IL12A, IL12B, IL13, IL13RA1, IL15, IL15RA, IL16, IL17A, IL18, IL19, IL1A, IL1B, IL1R1, IL2, IL20, IL23A, IL2RA, IL2RB, IL2RG, IL3, IL3RA, IL4, IL4R, IL5, IL6, IL6R, IL6ST, IL7, IL8, IL9, INSR, IRF1, IRF9, ISG15, JAK1, JAK2, JAK3, JUN, JUNB, KIT, MAP2K1, MAP2K2, MAP2K3, MAP2K4, MAP2K5, MAP2K6, MCL1, MERTK, MET, MMP2, MMP9, MPL, MYC, MYD88, NFKB1, NGFR, NOS2, NR3C1, OSM, PDGFRA, PDGFRB, PIAS1, PIAS2, PIK3C3, PIK3CA, PIK3CB, PIK3CD, PIK3R1, PRL, PRLR, PTEN, PTPN1, PTPN11, PTPN6, PTPRC, RELA, SH2B1, SMAD1, SMAD2, SMAD3, SMAD4, SMAD5, SMAD6, SMAD7, SOCS1, SOCS2, SOCS3, SOCS4, SOCS5, SPI1, SRC, STAM, STAT1, STAT2, STAT3, STAT4, STAT5A, STAT5B, STAT6, SUMO1, SUMO2, SUMO3, TP53, TYK2, TYRO3, VEGFA*
PI3K-AKT<br>n = 92	*AKT1S1, AKT2, AKT3, ATP6V1B2, BAD, BORCS6, BRF2, CAD, DDIT4, DEPTOR, DPYSL2, ERAS, FKBP5, FNIP2, FOXA2, FOXO1, FOXO3, FOXO4, GRB10, GSK3A, GSK3B, HTT, INPP4B, INPPL1, KIF1B, LAMTOR1, LAMTOR2, LAMTOR3, LAMTOR4, LAMTOR5, LARS1, MACC1, MAP4K3, MAPKAP1, MDM2, MIOS, MOB1A, MOB1B, NDRG1, NDRG2, NDRG3, NDRG4, NOP53, NOS1, NOS3, NPRL2, NR4A1, PARK7, PDPK1, PHLDA3, PI4KA, PIK3C2A, PIK3C3, PIK3CA, PIK3CB, PIK3CD, PIK3CG, PIK3R1, PIK3R2, PIK3R3, PIK3R4, PIK3R5, PPP1R10, PRKD2, PRR5L, PTEN, RANBP3, RHEB, RPS6KB2, RRAGB, SEMA4B, SGK1, SGK2, SGK3, SH3BP4, SPAG5, SYAP1, TBC1D7, TCL1A, THEM4, TNS2, TSC1, TSC2, WDR59, WNK1, WNK4, WWTR1, YAP1, YBX1, ZFP36L1, ZFYVE26*
NF-kappa B<br>n = 327	*AGER, AICDA, AIM2, ALOX5, APEX1, ARIH2, ATRAID, B2M, BACH2, BATF, BCL10, BCL11B, BCL6, BLK, BLNK, BMX, BTK, C1QBP, CABIN1, CARD11, CARD9, CBL, CBLB, CCL2, CCL5, CCR2, CD19, CD2, CD276, CD28, CD2AP, CD34, CD38, CD3E, CD40LG, CD44, CD46, CD63, CD74, CD79A, CD82, CD83, CD8A, CDCP1, CDIP1, CEACAM1, CEACAM5, CEBPA, CEBPB, CEBPD, CHUK,*

CIITA, CISH, CSF2RB, CTLA4, CXCL10, CXCL12, CXCR4, CXCR5, CYLD, DAPP1, DCLRE1C, DDX58, DHX58, EGR1, ENPP3, EOMES, ERC1, FAS, FASLG, FER, FLI1, FOXP3, FYB1, GATA1, GATA2, GATA3, GATA6, GDF15, GFI1B, GIMAP5, GPX1, GRK6, GZMA, GZMB, HAVCR1, HAVCR2, HCK, HCLS1, HMOX1, ICAM2, ICOS, ID3, IDO1, IFI16, IFIH1, IFNA1, IFNG, IKBKB, IKBKE, IKBKG, IKZF1, IKZF3, IL10, IL17A, IL1B, IL1RN, IL2, IL2RA, IL2RB, IL3, IL4, IL6ST, INPP5D, INPPL1, IRAK1, IRAK2, IRAK3, IRAK4, IRF1, IRF2, IRF3, IRF4, IRF5, IRF6, IRF7, IRF8, ITCH, ITGB2, ITK, JAK1, JAK2, JAK3, JUNB, KIT, KITLG, L1CAM, LAG3, LAT, LAT2, LCK, LCP1, LCP2, LGALS1, LGALS3, LGALS9, LILRB1, LITAF, LSP1, LYN, MAGEA3, MALT1, MAP3K14, MAP3K7, MAP4K1, MAVS, MECOM, MLANA, MNDA, MPL, MPO, MRTFA, MYB, MYD88, NCAM1, NCF1, NCF4, NCK1, NDRG1, NFATC1, NFATC2, NFATC3, NFATC4, NFIL3, NFKB1, NFKB2, NFKBIA, NFKBIB, NFKBIE, NFKBIZ, NLRP1, NLRX1, NOD1, NQO1, NR1D1, NR4A1, NT5E, OAS1, OTUD5, OTUD7B, OTULIN, PAWR, PAX5, PBX1, PDCD1, PDCD1LG2, PDLIM2, PECAM1, PELI1, PIAS1, PIAS3, PIAS4, PIM1, PIM2, PIM3, PIR, PLCG2, PPIA, PPP3CA, PPP3CB, PPP3CC, PRAME, PRDM1, PRKCQ, PRLR, PSIP1, PSMB8, PTGS1, PTGS2, PTK2B, PTPN2, PTPN22, PTPN6, PTPRC, PVR, RABGEF1, RAG1, REL, RELA, RELB, RIPK1, RIPK2, RIPK3, RIPK4, RNASEL, RSAD2, RUNX1, RUNX1T1, SARM1, SH2D1A, SH3KBP1, SHARPIN, SIRPA, SIVA1, SOCS1, SOCS2, SOCS3, SPI1, SPIB, STAM2, STAT1, STAT2, STAT3, STAT4, STAT5A, STAT5B, STAT6, STK17B, SWAP70, SYK, TAB1, TAB2, TAB3, TAL1, TANK, TAP1, TAP2, TAX1BP1, TBK1, TBKBP1, TBX21, TCF12, TCF3, TEC, THEMIS, TICAM1, TLR1, TLR2, TLR3, TLR6, TLR7, TLR8, TLR9, TNF, TNFAIP3, TNFRSF10B, TNFRSF10D, TNFRSF11A, TNFRSF12A, TNFRSF17, TNFRSF18, TNFRSF1A, TNFRSF1B, TNFRSF25, TNFRSF4, TNFRSF8, TNFRSF9, TNFSF11, TNFSF12, TNFSF13B, TNFSF15, TNFSF4, TNIP1, TOLLIP, TPT1, TRAF1, TRAF2, TRAF3, TRAF4, TRAF5, TRAF6, TRIB2, TRIM25, TXN, TYK2, TYROBP, UBD, UBE2N, USP15, VCAM1, VSIR, VTCN1, XRCC5, ZAP70, ZBTB16, ZBTB7B
