# Supplementary figures and images for: The Role of PD-L1 Expression in Prediction and Stratification of Recurrent or Refractory Extranodal Natural Killer/T-Cell Lymphoma
Source: Front Oncol. 2022 May 10;12:821918. doi: 10.3389/fonc.2022.821918 (PMC9128790; doi:10.3389/fonc.2022.821918)

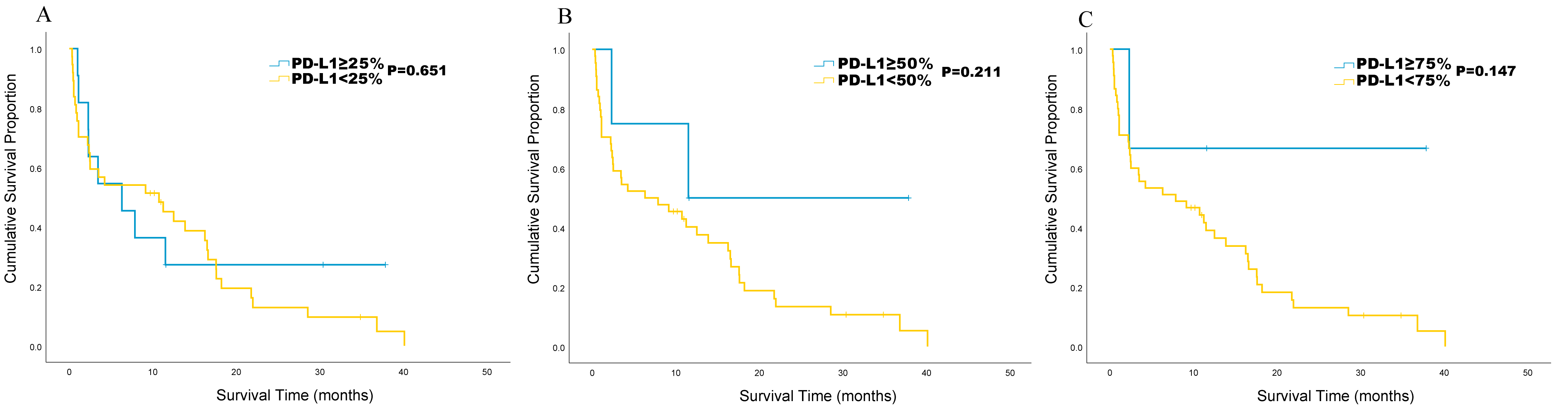

Supplement: Supplementary Figure 1 — Kaplan-Meier survival analysis for relapsed or refractory (RR) ENKTL patients with different cut-off values of PD-L1. (A) Overall survival (OS) by PD-L1 cut-off value of 25%; (B) OS by PD-L1 cut-off value of 50%; (C) OS by PD-L1 cut-off value of 75%. [file Image_1.tif]
